# A Case of Adrenal Crisis-Induced Stress Cardiomyopathy

**DOI:** 10.7759/cureus.14420

**Published:** 2021-04-11

**Authors:** Chad L Harris, Mazin Khalid, Arsalan Hashmi, Jacob Shani, Bilal A Malik

**Affiliations:** 1 Internal Medicine, Maimonides Medical Center, New York, USA; 2 Cardiology, Maimonides Medical Center, New York, USA

**Keywords:** takutsubo cardiomyopathy, addisons disease, adrenal insufficiency

## Abstract

We report a case of a 36-year-old male who presented to the emergency department with complaints of weakness. On presentation the patient was hypotensive, hyperkalemic, and hyponatremic. The patient experienced a sudden cardiac arrest in the computed tomography (CT) scanner moments after arrival. Electrocardiogram (EKG) demonstrated PR prolongation and widened QRS. Echocardiogram demonstrated a left ventricular ejection fraction of 26%-30% with evidence of severe hypokinesis of the mid antero-septal and inferior-septal segments of the left ventricle. CT of the chest, abdomen, and pelvis demonstrated hypoplastic/atrophic adrenal glands. Total cortisol level was undetectable by lab measurement. The patient was diagnosed with stress cardiomyopathy secondary to adrenal crisis. He was managed with hydrocortisone and eventually made a full clinical recovery and improvement in left ventricular ejection fraction. This article references the rarity of this phenomenon and its relevance to early clinical detection.

## Introduction

Stress cardiomyopathy, also known as Takotsubo cardiomyopathy, is characterized by transient ventricular dysfunction and wall motion abnormalities, leading to reduced left ventricular ejection fraction (LVEF), which usually develops after a high-stress state [1,2]. This phenomenon is most commonly observed in postmenopausal women and is believed to be secondary to catecholamine surge [3]. Stress cardiomyopathy secondary to adrenal insufficiency is extremely rare, with only two documented cases [4,5]. Here we report a case of a 36-year-old male with stress cardiomyopathy precipitated by adrenal crisis.

## Case presentation

**Figure 1:**
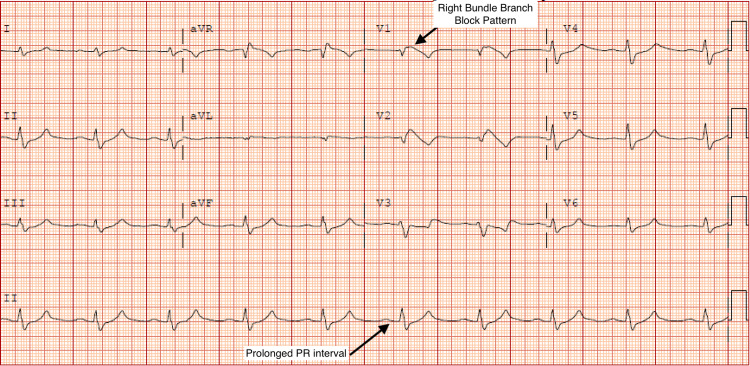
EKG showing sinus rhythm, PR prolongation, right bundle branch block pattern.

**Figure 2:**
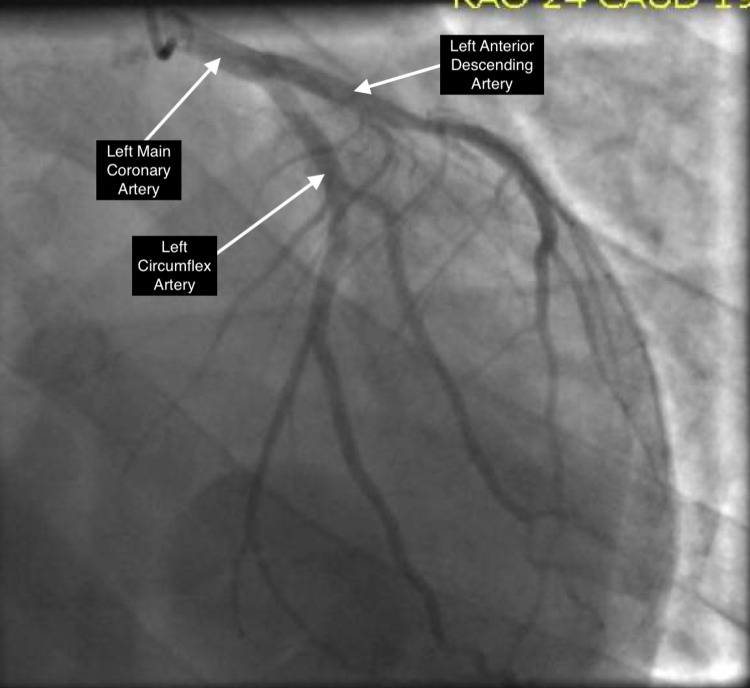
Coronary angiogram of the left coronary system from a right anterior oblique position with caudal angulation. It shows patent left anterior descending and left circumflex arteries.

**Figure 3:**
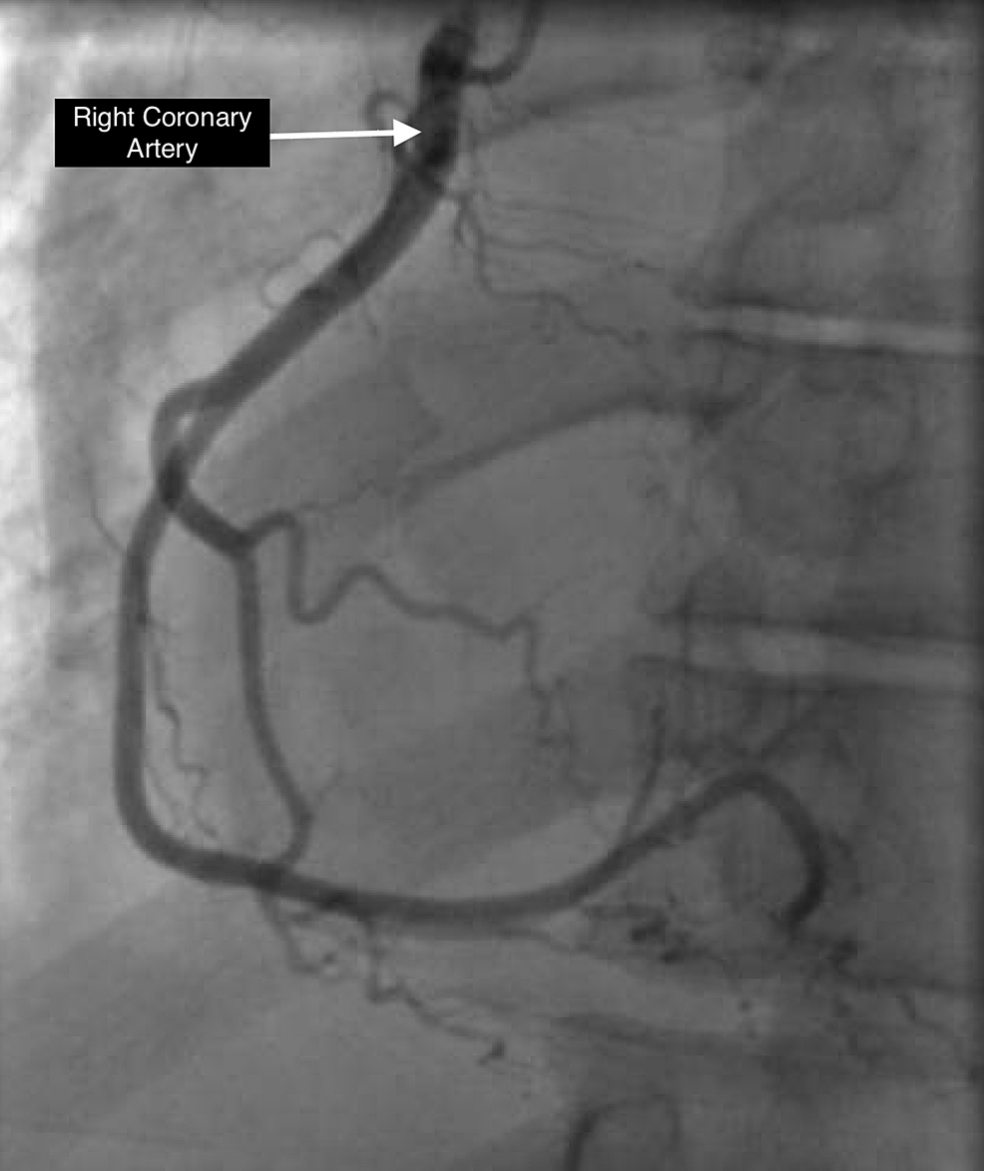
Coronary angiogram of the right coronary system in left anterior oblique position showing a patent right coronary artery.

A 36-year-old male with no past medical history presented to the emergency room after a syncopal event on the subway, prior to which he was feeling weak in all his extremities. On presentation, systolic blood pressure was in the low 80s; labs were significant for potassium of 8.8 mEq/l and sodium of 124 mEq/l. Initial troponin was zero but increased to a max of 0.92 ng/ml. Electrocardiogram (EKG) demonstrated a right bundle branch block pattern with coved ST elevation in right precordial leads, PR prolongation, and widened QRS, with no prior EKG for comparison (Figure 1). The patient suffered a cardiac arrest shortly after arrival. Rhythm at that time was consistent with pulseless electrical activity. Return of spontaneous circulation (ROSC) was achieved after six minutes. The patient was intubated, started on vasopressors, stabilized, and admitted to the intensive care unit (ICU). The patient was then started on continuous venovenous hemodialysis (CVVHD) for refractory hyperkalemia. Computed tomography (CT) of the chest, abdomen, and pelvis demonstrated hypoplastic/atrophic adrenal glands. Total cortisol level was undetectable by lab measurement, and adrenocorticotropic hormone (ACTH) was elevated to 261, concerning for a new diagnosis of primary adrenal insufficiency. The patient was started on stress dose hydrocortisone. Echocardiogram performed on admission demonstrated a LVEF of 26%-30% with evidence of severe hypokinesis of basal and mid inferior septum and mid antero-septal segments (Videos 1, 2). Cardiac catheterization revealed normal coronary arteries (Figures 2, 3).The patient eventually was titrated off vasopressors, extubated, and discharged on hydrocortisone and fludrocortisone. Repeat echocardiogram two weeks later demonstrated improvement of wall motion abnormalities with LVEF to 41%-45%.

**Video 1:** Transthoracic echocardiogram, parasternal long axis view, showing dilated left ventricle, reduced left ventricular systolic function, and hypokinesis in the mid anteroseptum and inferolateral segments.

**Video 2:** Transthoracic echocardiogram with contrast, apical three-chamber views, showing dilated left ventricle, reduced left ventricular systolic function, and hypokinesis in the mid and apical segments of both anteroseptum and inferolateral segments and apex.

## Discussion

Stress cardiomyopathy, also known as Takotsubo cardiomyopathy, was first described in 1990 in Japan and quickly gained recognition [6]. It is characterized by transient ventricular dysfunction and wall motion abnormalities leading to a reduction in the LVEF, which usually develops after a high-stress state [1,2]. This syndrome is most commonly observed in postmenopausal women, which is believed to be secondary to the effects of catecholamine surge on the myocardium [3]. The presentation can vary from acute chest pain to fulminant heart failure. EKG findings and cardiac biomarkers are consistent with the acute coronary syndrome with an unremarkable angiogram. Echocardiogram generally demonstrates a pattern of apical ballooning with spared basal segments, but other patterns such as apical/inverted Takatsubo with spared apex and hypo/akinetic base exist. Multiple diagnostic criteria exist for this syndrome. Most include hypokinesis, dyskinesis, or akinesis of the left ventricular segment, no obstructive coronary artery disease on angiography, and new ECG abnormalities or elevated cardiac troponin. Ventricular dysfunction resolves in nearly all documented cases [7].

Addison's disease, also known as adrenal insufficiency, can also have variable presentations, ranging from simple gastrointestinal symptoms to hemodynamic compromise and shock [8,9]. Metabolic or infectious derangements usually precipitate catastrophic events that a normal hormonal stress response would otherwise compensate.

Two cases of Addison's stress-induced cardiomyopathy have been reported in the literature; however, this case is unique in several aspects [4,5]. One case did report a similar adrenal crisis presentation; however, our case is unique in which this patient had a cardiac arrest secondary to this adrenal crisis. He required intubation, vasopressors, and continuous venovenous hemodialysis (CVVHD). Additionally, the patient did not have a previous diagnosis of adrenal insufficiency and presented in acute adrenal crisis. Wall motion abnormalities and decreased LVEF had partially resolved at two weeks follow-up. Initial EKG demonstrated a right bundle branch block pattern with coved ST elevation in right precordial leads concerning for a Brugada-like pattern; however, these abnormalities normalized after correction of the hyperkalemia. Although it is possible that the patient arrested secondary to severe hyperkalemia and that this arrest may have contributed to the development of cardiomyopathy, this case still presents a unique presentation that has been reported rarely in the literature.

## Conclusions

Stress cardiomyopathy secondary to adrenal insufficiency is extremely rare, with only two documented cases. Here we report a case of a 36-year-old male with stress cardiomyopathy secondary to adrenal crisis with associated electrocardiogram and echocardiogram findings. After treatment of the patient’s new diagnosis of adrenal insufficiency, he made a full recovery and regained his systolic function. This patient's presentation's unique nature reinforces the necessity to maintain a high index of suspicion of adrenal crisis in hypotensive and hyperkalemic patients. Early identification may prevent further occurrences of this phenomenon.
